# Unsupervised Deep Learning Registration of Uterine Cervix Sequence Images

**DOI:** 10.3390/cancers14102401

**Published:** 2022-05-13

**Authors:** Peng Guo, Zhiyun Xue, Sandeep Angara, Sameer K. Antani

**Affiliations:** Lister Hill National Center for Biomedical Communications, National Library of Medicine, National Institutes of Health, 8600 Rockville Pike, Bethesda, MD 20894, USA; zhiyun.xue@nih.gov (Z.X.); sandeep.angara@nih.gov (S.A.); santani@mail.nih.gov (S.K.A.)

**Keywords:** unsupervised registration, deep learning, uterine cervix cancer, image registration, image segmentation, automated visual evaluation, transformer

## Abstract

**Simple Summary:**

A sequence of images can be taken after the application of acetic acid during a colposcopic examination of the uterine cervix to capture the dynamic visual variations due to the aceto-whitening reaction on the cervical epithelium. Automated analyis of these changes require spatial alignment of the apparent change in the cervix location in the image sequence due to patient movement or imaging device repositioning. We developed a new self-supervised RGB-colored deep learning-based image registration method to automatically align the images that does not require a manually-provided reference standard. We also fine-tuned a transformer-based segmentation network to evaluate the result of our registration method which achieved 12.62% higher in Dice/IoU scores in cervix boundary detection compared to the unregistered images.

**Abstract:**

During a colposcopic examination of the uterine cervix for cervical cancer prevention, one or more digital images are typically acquired after the application of diluted acetic acid. An alternative approach is to acquire a sequence of images at fixed intervals during an examination before and after applying acetic acid. This approach is asserted to be more informative as it can capture dynamic pixel intensity variations on the cervical epithelium during the aceto-whitening reaction. However, the resulting time sequence images may not be spatially aligned due to the movement of the cervix with respect to the imaging device. Disease prediction using automated visual evaluation (AVE) techniques using multiple images could be adversely impacted without correction for this misalignment. The challenge is that there is no registration ground truth to help train a supervised-learning-based image registration algorithm. We present a novel unsupervised registration approach to align a sequence of digital cervix color images. The proposed deep-learning-based registration network consists of three branches and processes the red, green, and blue (RGB, respectively) channels of each input color image separately using an unsupervised strategy. Each network branch consists of a convolutional neural network (CNN) unit and a spatial transform unit. To evaluate the registration performance on a dataset that has no ground truth, we propose an evaluation strategy that is based on comparing automatic cervix segmentation masks in the registered sequence and the original sequence. The compared segmentation masks are generated by a fine-tuned transformer-based object detection model (DeTr). The segmentation model achieved Dice/IoU scores of 0.917/0.870 and 0.938/0.885, which are comparable to the performance of our previous model in two datasets. By comparing our segmentation on both original and registered time sequence images, we observed an average improvement in Dice scores of 12.62% following registration. Further, our approach achieved higher Dice and IoU scores and maintained full image integrity compared to a non-deep learning registration method on the same dataset.

## 1. Introduction

According to the World Health Organization (WHO), cervical cancer is the fourth-most-common cancer in women, with an estimated 570,000 new deaths in 2018 [1]. Since the disease is treatable, early detection/screening is critical for cervical cancer control. Screening methods include the Pap test (cytology), Human Papillomavirus (HPV) detection, colposcopy, visual inspection with Lugol’s Iodine (VILI), and visual inspection with acetic acid (VIA). VIA, being simple and inexpensive, is commonly used in middle- or low-resource regions instead of colposcopy. The VIA test is conducted by visually examining the cervix appearance with naked eyes after applying dilute (3–5%) acetic acid to the speculum-exposed cervix during a gynecological exam. The temporary whitening effect of the acetic-acid-applied region is considered an indicator of possible HPV infection [2]. However, VIA has been noted for inadequate performance due to highly subjective human impression decision-making [3] and the subtle differences between different stages of abnormalities.

Previously, we have proposed several deep-learning-based methods using multiple datasets for cervical precancer detection [4,5,6,7]. We grouped them under the moniker automated visual evaluation (AVE) to describe the set of techniques that have shown their potential to collectively serve as an adjunct to the VIA screening approach. One of the steps in these AVE methods is to use an object detection network either as a base network or as a cervix region bounding box generator to localize our regions of interest (ROIs). While bounding boxes are convenient for AVE, they are coarse annotations that may contain a significant amount of irrelevant information, such as clinical instruments (the speculum and swab) and other non-cervical tissues (vaginal walls). To alleviate this issue, we investigated deep learning methods of Mask R-CNN and Mask^x^ R-CNN to automatically segment the cervix region [8] and the os [9] region. These segmentation models were trained using multiple datasets and achieved satisfactory performance across datasets.

There has been growing interest in the use of image sequences during a colposcopic examination for cervix diagnostic assessments [10,11]. In this process, images are acquired at regular time intervals following the application of acetic acid on the cervix. Image sequences are helpful for visualizing the aceto-whitening process, which could provide additional diagnostic information. However, due to patient and camera movement, the appearance of the primary regions of interest (ROIs), such as the cervix and the os, may vary significantly from frame to frame in terms of focus, location, size, shape, and illumination. In addition, maintaining the color information of these images is potentially helpful for capturing the dynamic reactions of the aceto-whitening effect on the cervical epithelium. This presents a significant need for aligning these ROIs in full color toward aiding the quality and accuracy of the subsequent automated analyses.

In this study, we propose an unsupervised deep learning image registration approach for three-color-channel cervix images. Our network is trained using image frames extracted from 2 min colposcopy image sequences. Each time series is captured during a routine colposcopy exam in a clinical setting using a colposcope manufactured by DYSIS Medical (DYSIS Medical Inc., Natick, MA, USA). In our unsupervised strategy, we developed a customized architecture consisting of three branches, one for each color channel—red (R), green (G), and blue (B). Each branch uses a feature-extracting CNN, and a spatial transform layer [12]. Due to the unavailability of registration ground truth, we use a segmentation network built over a base network of DeTr (Object Detection with Transformer) [13] to evaluate our registration performance. The DeTr-based segmentation model is trained using three datasets [8,9] collected in different geographical regions using different imaging devices. These three datasets have large intra- and inter-dataset variability regarding color, illumination, position, size, and other related visual factors. In addition, there are wide variations among images in these datasets with respect to image quality (focus, specular reflection, artifacts, and camera zoom level), which pose significant challenges for the segmentation task.

There is extensive work in the literature on medical image registration that includes both learning-based and non-learning-based methods. The non-learning-based methods include statistic-based metric mapping [14,15,16,17], distance metric mapping [18], and differentiation-based deformation [19]. The learning-based methods [20,21,22,23] have demonstrated desirable performance in the registration of brain and breast MRI. Non-learning-based methods rely on the repetition of similarity in handcrafted features across all datasets, thereby suffering in generalizability. In contrast, the performance of learning-based methods depends on the quantity and quality of the ground truth and the variety and size of the training dataset. The literature also includes unsupervised deep-learning-based methods [24,25,26] developed for biomedical image registration tasks. In [24], a CNN regressor was used with the loss of a similarity metric measurement and achieved promising results on the MNIST dataset. Later, using a similar architecture in [26], satisfactory performance was observed adding a spatial variation loss with fMRI. Although the methods’ performances on full RGB color images registration were not presented, we were inspired by the proposed architectures of using an encoder–decoder CNN. In this study, we used a more efficient encoder–decoder architecture with fewer parameters and extended the network input from a single grayscale channel to R, G, and B color channels, respectively.

There are a limited number of studies on cervix region segmentation in photographic images of the uterine cervix. A compact deep learning architecture was applied [27] on 32- × 32-pixel patches as an intermediate stage for classification. However, quantitative results were not reported. In [28], the authors reported an average Dice score of 0.67 using a convolutional neural network (CNN) model that was implemented over a cervix dataset collected by MobileODT (Kaggle Dataset, Tel Aviv, Israel). In [28,29], a Mask R-CNN was applied to cervix segmentation tasks and obtained (Dice, IoU) scores of (0.865, 0.769), respectively. In our previous study, we made efforts in cervical landmark segmentation and achieved robust performance (highest Dice, IoU: 0.947, 0.901, respectively) across multiple image datasets [8,9]. In this paper, we use the same datasets as in [8,9] to evaluate our new segmentation method and compare the results with our previous best performance.

We report the development of an unsupervised deep learning approach for RGB cervix image registration. To evaluate the registration output, we fine-tuned an object detection DeTr model for a segmentation task to automatically generate region masks for the cervix for each frame in the registered dynamic image sequence. Next, we computed the Dice score of the masks for every frame in the sequence with the first frame—which serves as a reference. A high Dice score would suggest that the registration was good. An upper bound on the performance would be controlled by the error rate of the cervix segmentation algorithm. We also compared our registration approach with another non-deep-learning, conventional registration method on the same dataset and obtained higher measurements.

The rest of the paper is organized as follows: Section 2 describes the two proposed deep learning architectures: the color image registration network and the fine-tuned segmentation network; Section 3 presents the experiments, the results, and the discussion; and Section 4 concludes the paper.

## 2. Materials and Methods

### 2.1. Datasets

Four datasets were used in this study, which are described below. All datasets were deidentified at source and are permitted for research use. The first three datasets, viz. A, B, and C, have been used in our previous study [8,9] for landmark segmentation. They are used in this study to train the proposed segmentation models. The DYSIS dataset is used to train and evaluate the registration models.

#### 2.1.1. CVT Dataset

**Figure 1:**
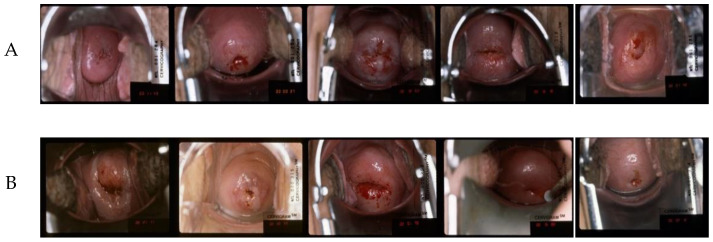

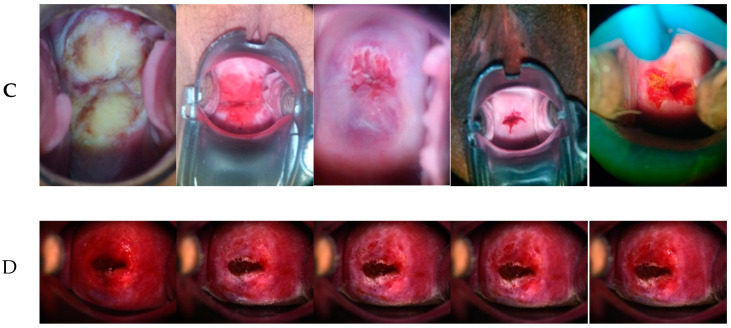
Example images from the above four datasets. The 1st row demonstrates the CVT (**A**) dataset; the 2nd row shows samples from the ATLS (**B**) dataset; Kaggle (**C**) images are presented in the 3rd row; and images from the DYSIS (**D**) dataset are provided in the 4th row; the frame indexes are 1st, 4th, 8th, 13th, and 17th in the time sequence, from left to right, correspondingly.

This dataset is referred to as set A (example images shown in Figure 1, row 1). The images were taken as part of the Costa Rica Vaccine Trial (CVT) conducted by the NCI [30,31]. After receiving the HPV16/18 vaccines, women participating in the study were referred for colposcopy if the cervical lesion persisted or if they had high-grade cytology at any time during the trial. The cervical images of women referred for colposcopy were acquired (with permission obtained during enrolment in the study) on photographic film and digitized. The ground truth segmentation masks of all the images in this dataset were annotated by an expert gynecologist.

#### 2.1.2. ALTS Dataset

The images in this dataset, denoted as set B (example images shown in Figure 1, row 2), were captured as part of the atypical squamous cells of undetermined significance/low-grade squamous intraepithelial lesion (ASCUS/LSIL) Triage Study (ALTS) [32]. The study aimed to assess observer variability in visual interpretation of cervical abnormalities. In this dataset, we have multiple ground truth mask labels as the images were annotated by several medical experts in cervical oncology. So, we took their union to obtain the final ground truth for each image [8,9].

#### 2.1.3. Kaggle Dataset

This dataset, referred to as set C (example images shown in Figure 1, row 3), was made publicly available as part of the data hosted on Kaggle for the “Intel & MobileODT Cervical Cancer Screening Competition” [33]. The images were acquired using a smartphone-based digital cervical imaging device. The image annotations include cervix region contours, which were made available in [28]. A large quality variation was observed in this dataset, including motion blur, presence of the speculum, reflection, poor lighting, and poor camera positioning, among other flaws (Figure 1).

#### 2.1.4. DYSIS Dataset

The images in this dataset, called set D (example images shown in Figure 1, row 4), were captured during routine colposcopy examinations. During each visit, a series of 17 images was acquired, from before to after the application of acetic acid, while visualizing the cervix using the DYSIS digital colposcope [10]. Variations are observed in the images in the dataset in cervix positioning and appearance, lighting intensity, image sharpness, and color.

### 2.2. Dataset Split

Datasets A, C, and D were split into training, validation, and test partitions with an approximate ratio of 7:1:2 to train the segmentation network. Dataset B was only used for training the network since the unique deidentified patient ID associated with each image is not available. Thus, splitting the dataset could result in data leakage. In Table 1, we summarize the quantitative details of the datasets.

### 2.3. Registration Network

**Figure 2:**
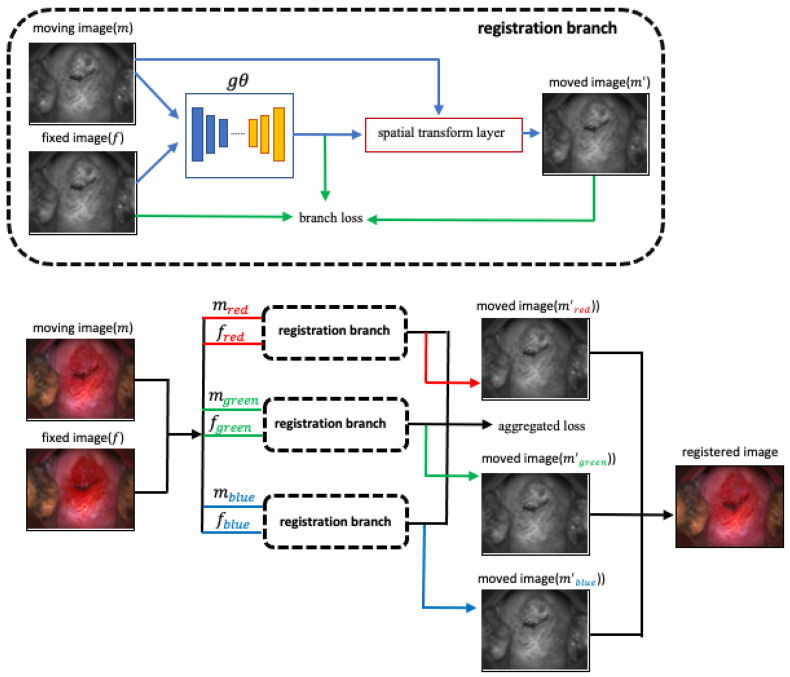
Network structure. To register an RGB color image, we use three branches, each of which takes one of the R, G, and B channels, of an image frame as the input, respectively. The input of each branch consists of two channels, a moving image and a fixed image, that are randomly sampled from the same time sequence. The architectural designs of each branch are the same, and the losses from each branch are aggregated and backpropagated.

Our unsupervised registration network, shown in Figure 2, consists of three branches. Each branch has a CNN architecture for feature extraction, a spatial transform unit, and a loss computation module. We treat each RGB color image as a split of R channel, G channel, and B channel image, respectively. Each of the R, G, and B channels is trained using one branch of the registration network, and their loss is aggregated and backpropagated. The network takes a batch of two images as input, called f and m to represent “fixed” and “moving”. These are the single-channel R, G, or B channel images randomly selected from a single sequence of 17 images. After passing through the convolutional feature extraction module g, image m is warped to $m^{\prime }$ using a spatial transformation layer, thereby enabling the model to evaluate the similarity of f and m and update the network weights gθ. The input of each branch is of size 1024 × 768 × 2, and we use Leaky ReLU activations with a convolutional kernel size of 3 × 3. The gθ consists of an encoder and a decoder of [16, 32, 32, 32, 32, 32, 32, 16] channels.

Losses: We use the similarity loss and the local spatial variation loss, discussed in [26], to penalize the difference in appearance and retain the local spatial smoothness of the warped image.

Data augmentation: To train our deep learning network model, we use data augmentation techniques to upsample each image sequence for each training period. The list of image augmentation techniques applied to input images is color shift, contrast changing, image sharpening, and Gaussian noise. We do not apply any augmentation technique that is related to spatial transformation, such as the affine transform, for training the registration algorithm.

Training scheme: We train the network per image sequence since ROI misalignment occurs within the group of images. In each training period, images in the same image sequence are shuffled and iteratively trained in one epoch. We use a batch size of one containing two randomly selected images, labeled as the “fixed” image and the “moving” image, for input. The next training period starts when all the frames/their augmentations are trained from the current time sequence.

### 2.4. Segmentation Network

**Figure 3:**
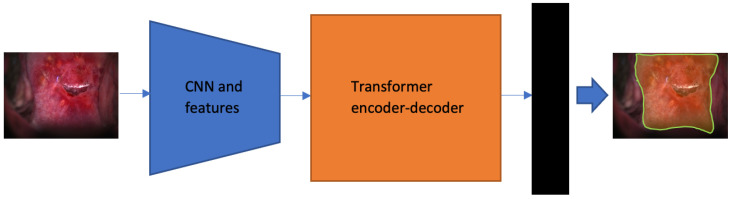
The DeTr-based model we constructed for segmentation. The box in black color represents the used mask head that is a multi-perceptron-layer (MPL) consisting of an 8-channel 1 × 1 fully convolutional module. In all, 3 encoder layers, 3 decoder layers, and 4 attention heads are used in the transformer units, and due to our target quantity limitation, we reduce the number of object query slots to 20 compared to that used in [13].

The vision transformer (ViT) [34] is an architecture inherited from Natural Language Processing [35] research and applied to image classification. It uses raw image patches as input instead of strings of textual characters. Deep learning architectures using transformers, such as the DeTr [13], have exhibited comparable results to non-transformer techniques for object detection. It is the first fully end-to-end object detector that uses a simple architecture consisting of convolutional neural networks (CNNs) and transformer encoders–decoders. The architecture consists of a backbone network for extracting features and a transformer encoder–decoder module followed by prediction heads (Figure 3). The encoder–decoder module is constructed with 3 encoder layers, 3 decoder layers, and 4 attention heads. The feedforward network (FFN) consists of 256 layers, with an embedding size of 128. We employ 20 object query slots, considering the object number in each image of our dataset. The matching loss, the bounding box loss, and the GIoU [36] loss are weighted equally. We apply the stage-wise training strategy presented in [8]: train a DeTr based object detection network in the first stage; then in this pre-trained model, replace the bounding box prediction heads with a mask prediction head and train the network with mask ground truth. We use this segmentation network to extract cervix region boundaries in both original images and registered images to evaluate the performance of our registration network.

## 3. Results

### 3.1. Implementation Details

To train the registration network, we resize each image to 1024 pixels on the longer edge and maintain the aspect ratio. The training is carried out on high-performance computers powered with four Nvidia Tesla V100 graphical processing units (GPU) with a batch of 1. The algorithm is trained using Keras (Tensorflow as the backend) for 420 epochs, with 1000 steps in each epoch. The average training period per epoch is around 548 s.

To train the segmentation network used for evaluating the registration results, we resize the images such that their longer edge is 600 pixels with the aspect ratio maintained. The training is carried out using the same GPUs as the registration training. ResNet50 is used as the backbone for feature extraction. The algorithm is trained using Pytorch for 50 epochs, with 1000 steps in each epoch, initializing the learning rate at 0.001 and decaying by 10 times at epoch 20. We use a batch size of 8, and the weight decay factor and momentum are set to be 0.0001 and 0.9, respectively. The average training period per epoch is around 298 s.

### 3.2. Experiment Results

Our evaluation of the registration performance is performed in two steps: (1) employ the trained segmentation network to generate cervix regions in both the original and the registered images; and (2) compare the mask predictions on the registered images and original images. This approach is used to overcome the deficit of a ground truth evaluation dataset. The segmentation performance is evaluated in advance to ensure that our algorithm delivers robust performance in the time sequence images. In addition, we conduct a qualitative visual inspection of all the registered images and present our observations.

#### 3.2.1. Visual Impression—Segmentation

**Figure 4:**
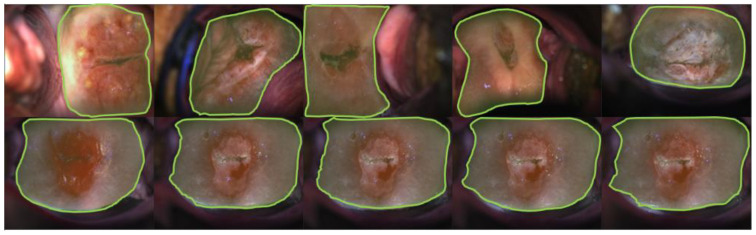
The segmentation predictions on test images of dataset D. Green lines mark the predicted boundaries. The 5 images in the top row are sample results randomly selected across the test dataset. Images in the bottom row are segmentation predictions from a single same-time sequence (one patient). The frame indexes are 1st, 4th, 8th, 13th, and 17th in the time sequence, from left to right, correspondingly.

We train the object detection network using the bounding box ground truth from datasets A, B, and C. The best model is selected considering the validation performance. Next, we train the segmentation network with the replaced mask head using the mask ground truth from the same dataset. We perform the test on the test splits of datasets A, B, C, and D. As shown in Figure 4 (bottom row), the predicted boundaries on dataset D images are mostly enclosing the cervix region, our ROIs. The borderlines are predicted with the same morphological patterns as compared with the ones in datasets A, B, and C images. We present the quantitative results for datasets A and C in Table 2. Note that our dataset B is only used as a training set and dataset D lacks ground truth. Therefore, the quantitative evaluation for these two datasets is not presented.

#### 3.2.2. Quantitative Measurements—Segmentation

As shown in Table 2, the proposed DeTr-based segmentation network achieves the best (Dice, IoU) score on dataset A, of (0.938, 0.885), and the best score on dataset C, of (0.917, 0.870). This performance is comparable with the corresponding scores of (0.945, 0.897) and (0.916, 0.863) that we obtained in [8]. Our segmentation approach in this study achieves performance that is comparable with that of the previous method in dataset C. In addition, our approach achieves a higher average Dice/IoU score in dataset A than in dataset C. We hypothesize that the images in dataset C are inherently more complex or there might be an insufficient number of images for representing the variety in cervix appearance.

#### 3.2.3. Visual Impression—Registration

**Figure 5:**
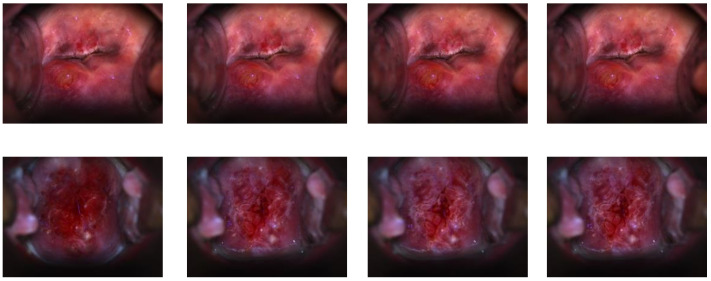
Registered time sequence image samples. Images in the top and bottom rows demonstrate consecutive images in a time sequence. The 1st image on the left of each row is the fixed one, which is the 1st image in the sequence, and each image toward the right side in each row demonstrates the 6th, 12th, and 17th registered sample in that time sequence.

To test the registration network, we used the first image of each time sequence as the reference image and registered each of the succeeding images (moving images) in the time sequence to that image. Then, we obtained a “registered” time sequence consisting of the reference image plus the network output of every moving image in time order. We visually examined our registered images for a qualitative assessment of performance. As shown in Figure 5, we found that our approach retained both the color and the morphological information of the cervix. In addition, the alignment is relatively stable under major or subtle color or texture variations generated due to the aceto-whitening effect.

#### 3.2.4. Quantitative Measurements—Registration

**Figure 6:**
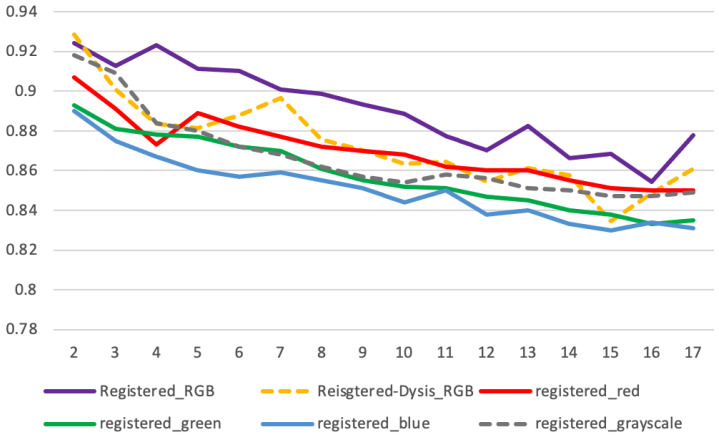
Dice (*y*-axis) measurement between the 2nd, 3rd, …, 17th (*x*-axis) image and the 1st fixed image in each sequence, using the registration algorithm by us (purple, legend: Registered_RGB) and by DYSIS (orange dash, legend: Registered-Dysis_RGB).

Apart from visual examination, we conducted a quantitative evaluation by comparing the segmentation mask prediction between the reference image and the registered images. We measured and compare: (1) the Dice score of the prediction masks of the original unregistered images and (2) the Dice score of the prediction masks of the registered images. The results are presented in Table 3. We observed an average Dice score of 0.792 in the unregistered images of all the tested original time sequences, and our registration approach achieved an average Dice score of 0.892 in the registered time sequences. We obtained an average improvement of 12.6% in the Dice score of segmentation of the registered over the unregistered sequence images. We also conducted experiments using single-color images, and in each experiment, the registration network consisted of only one “register branch” (Section 2.2). The network was trained using red-channel/green-channel/blue-channel or grayscale images, and we compared their single-channel registration output with those of RGB images (presented in Table 3 and Figure 6). We obtained average Dice scores of 0.869/0.858/0.850/0.866 using red/green/blue/grayscale images, respectively. We also noticed that the Dice scores in both unregistered and registered time sequence images drop over time. This indicated that more feature variation regarding cervix color and textures might be involved during the development of aceto-whitening effects, and the variation brings more challenges for both the segmentation and registration tasks.

**Figure 7:**
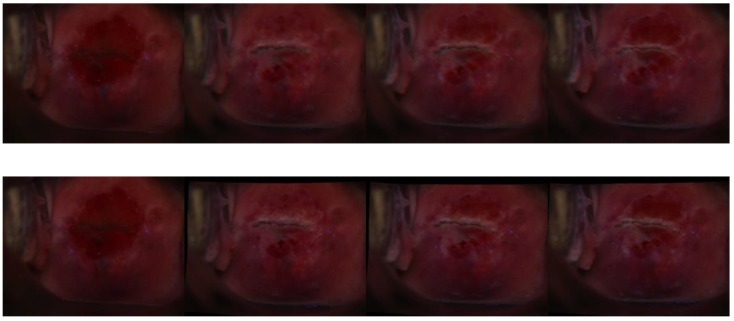
Registered time sequence image sample comparison between two methods. Images in the top row are from a time sequence registered by our approach; and the images in the bottom row are the same time sequence as registered by DYSIS [10]. The 1st image on the left of each row is the fixed image, and each image toward the right side in each row demonstrates the 6th, 12th, and 17th registered sample in that time sequence. Image distortion and defects can be observed in the bottom-row images, on edges and corners.

Further, we compared our registrations with the DYSIS time sequence registration [11] that uses a non-deep learning approach. We applied our segmentation approach to the registered time sequences and computed the average Dice scores for each indexed registered frame. From the results (Figure 6), we observed that our registration methods achieve a marginally higher average Dice score (0.89 vs. 0.87). We also compared our registration with the conventional methods developed by DYSIS [10] via visual examination of each time sequence. It can be observed that the conventional method involved much image distortion and image defects (blank space filled with black pixels), which could have been caused by affine transformation, pixel interpolation, etc. In the same time sequence, our method maintains image integrity, as shown in the example images in Figure 7. It remains to be seen whether, and to what extent, such differences would impact the outcomes of an AVE algorithm.

#### 3.2.5. Limitations

Although our registration approach provides better cervix alignment and retains color features of the ROIs, we observed that in some cases, there is subtle blurriness on cervix edges and over air bubbles in the aceto-whitened area. Most of these examples are of low sharpness, which might be caused by factors such as human motion, changes in lighting condition, and reflection, which could lower the image quality.

## 4. Conclusions

In this work, we have presented an unsupervised deep learning method for registering cervical images in time sequences and a robust segmentation network based on DeTr, a transformer-based object detection method for evaluating the registration. Using multiple datasets, we trained the cervix region segmentation network in a stage-wise scheme where we fine-tune an object detection model into a segmentation model by replacing the bounding box head with a mask prediction head. We further evaluated the segmentation network on multiple datasets that were also used in our previous study and achieved comparable performance. From the segmentation results, we can see that our current segmentation model overperforms the previous segmentation model on a challenging dataset. Further, we trained an unsupervised, channel-independent registration network that consists of a CNN module followed by a spatial transform module. With the robust performance of our segmentation network on the time sequence dataset, we evaluated the registration performance using the predicted segmentation masks. We achieved a higher average Dice score on the registered time sequences than that on the original unregistered time sequences.

Accurate alignment of cervix regions in a time sequence can help in landmark tracking and may assist in automated longitudinal analysis for AVE. It can also help retrieve clinically significant information, such as lesion presence, squamous columnar junction (SCJ) location, and aceto-whitening area, for visual analysis in a dynamic routine. In a future study, we plan to continue optimizing the algorithm for achieving more robust registration performance.

## Figures and Tables

**Table 1:** Quantitative details of Datasets A, B, C, and D.

Dataset Label	Image Size	Number of Images		
		Training	Validation	Test
A	1200 × 800	2447	350	601
B	1200 × 800	939	0	0
C	900 × 1200	1268	180	502
D	1024 × 768	210 × 17	30 × 17	60 × 17

**Table 2:** Results of testing our segmentation results on datasets A and C. Note that the previous method [8] referenced here is a Mask^x^ R-CNN model with a base net of ResNet50 architecture in the feature pyramid and the region proposal network. The measurements use Dice/IoU scores.

Tested Dataset	Testing Measurements (Dice/IoU)	
	This Study	Prior Work [8]
A	0.938/0.885	0.945/0.897
C	0.917/0.870	0.916/0.863

**Table 3:** Average Dice scores of each pair of fixed and succeeding images in the unregistered time sequence (left section) and the registered time sequence (right section). The numbers 2nd, 3rd, 4th, …, 17th denote the Dice scores calculated between the prediction mask on the nth image and the 1st image in that time sequence; see the line plot in Figure 6.

Unregistered				Registered (RGB/Red/Green/Blue/Grayscale)			
2nd	0.908	10th	0.782	2nd	0.924/0.907/0.893/0.890/0.918	10th	0.889/0.868/0.852/0.844/0.854
3rd	0.878	11th	0.773	3rd	0.913/0.891/0.881/0.875/0.909	11th	0.878/0.862/0.851/0.850/0.858
4th	0.852	12th	0.770	4th	0.923/0.873/0.878/0.867/0.884	12th	0.870/0.860/0.847/0.838/0.856
5th	0.840	13th	0.748	5th	0.911/0.889/0.877/0.860/0.880	13th	0.883/0.860/0.845/0.840/0.851
6th	0.802	14th	0.733	6th	0.910/0.882/0.872/0.857/0.872	14th	0.866/0.855/0.840/0.833/0.850
7th	0.803	15th	0.740	7th	0.901/0.877/0.870/0.859/0.868	15th	0.869/0.851/0.838/0.830/0.847
8th	0.801	16th	0.739	8th	0.899/0.872/0.861/0.855/0.862	16th	0.854/0.850/0.833/0.834/0.847
9th	0.767	17th	0.741	9th	0.893/0.870/0.855/0.851/0.857	17th	0.878/0.850/0.835/0.831/0.849

## Data Availability

For image data used in this study, please send special request to Mark Schiffman (NCI) at schiffmm@exchange.nih.gov.
